# A Survey of Compound Heterozygous Variants in Pediatric Cancers and Structural Birth Defects

**DOI:** 10.3389/fgene.2021.640242

**Published:** 2021-03-22

**Authors:** Dustin B. Miller, Stephen R. Piccolo

**Affiliations:** Department of Biology, Brigham Young University, Provo, UT, United States

**Keywords:** pediatric cancer, structural birth defect, germline variants, compound heterozygous variants, genetic analysis of complex diseases, trios

## Abstract

Compound heterozygous (CH) variants occur when two recessive alleles are inherited and the variants are located at different loci within the same gene in a given individual. CH variants are important contributors to many different types of recessively inherited diseases. However, many studies overlook CH variants because identification of this type of variant requires knowing the parent of origin for each nucleotide. Using computational methods, haplotypes can be inferred using a process called “phasing,” which estimates the chromosomal origin of most nucleotides. In this paper, we used germline, phased, whole-genome sequencing (WGS) data to identify CH variants across seven pediatric diseases (adolescent idiopathic scoliosis: *n* = 16, congenital heart defects: *n* = 709, disorders of sex development: *n* = 79, ewing sarcoma: *n* = 287, neuroblastoma: *n* = 259, orofacial cleft: *n* = 107, and syndromic cranial dysinnervation: *n* = 172), available as parent-child trios in the Gabriella Miller Kids First Data Resource Center. Relatively little is understood about the genetic underpinnings of these diseases. We classified CH variants as “potentially damaging” based on minor allele frequencies (MAF), Combined Annotation Dependent Depletion scores, variant impact on transcription or translation, and gene-level frequencies in the disease group compared to a healthy population. For comparison, we also identified homozygous alternate (HA) variants, which affect both gene copies at a single locus; HA variants represent an alternative mechanism of recessive disease development and do not require phasing. Across all diseases, 2.6% of the samples had a potentially damaging CH variant and 16.2% had a potentially damaging HA variant. Of these samples with potentially damaging variants, the average number of genes per sample was 1 with a CH variant and 1.25 with a HA variant. Across all samples, 5.1 genes per disease had a CH variant, while 35.6 genes per disease had a HA variant; on average, only 4.3% of these variants affected common genes. Therefore, when seeking to identify potentially damaging variants of a putatively recessive disease, CH variants should be considered as potential contributors to disease development. If CH variants are excluded from analysis, important candidate genes may be overlooked.

## Introduction

Each year in the United States, ∼3.0% of babies are born with a structural defect, and ∼11,050 children under the age of 15 are diagnosed with pediatric cancer (Lupo et al., 2019; American Cancer Society, 2020). Researchers are working to understand the genetic causes of these diseases, now often via whole-genome sequencing (WGS) (Zhang et al., 2015; Gröbner et al., 2018; Lupo et al., 2019). When researchers analyze DNA sequencing data, the parent of origin for each nucleotide is often unknown (Choi et al., 2018). While this is not an issue when looking at heterozygous variants in dominantly inherited diseases or when identifying homozygous alternate (HA) variants in recessively inherited diseases, it is problematic when looking at more complex inheritance patterns, such as compound heterozygous (CH) variants (Sanjak et al., 2017). Under the assumptions of recessive disease inheritance, in the majority of cases, a single heterozygous variant in a gene does not result in decreased fitness of an organism (Morrill and Amon, 2019). However, CH variants—which consist of alternate alleles positioned at different loci within a gene on opposite homologous chromosomes—may result in no functional copies of the associated proteins and a decrease in overall fitness. CH variants have been observed in many pediatric diseases. For example, pathogenic CH variants in the *ASXL3* gene can lead to a disruption in ASXL3 protein expression, potentially contributing to the development of congenital heart disease (Fu et al., 2020). Additionally, potentially damaging CH variants have been identified in many cancer predisposition genes, across various pediatric cancer types (Miller and Piccolo, 2020a). Although the importance of assessing CH variants in pediatric diseases has been established, researchers may ignore these types of variants in genome-wide studies due to additional steps required to identify and interpret them; one of these steps is haplotype estimation via computational phasing. It has been shown that using a population-based, haplotype reference panel and/or trio-based samples can improve phasing as much as 10-fold (Choi et al., 2018); however, this process is computationally expensive, and current phasing algorithms require specific file formats or that reads be aligned to a specific reference genome and be free of multi-allelic positions. These requirements may deter some researchers from performing phasing—and thus identifying CH variants.

Prior studies involving CH variant identification have focused primarily on individual diseases, one or a few samples, or specific genes (Miller and Piccolo, 2020a). Furthermore, little is known about how to filter and classify CH variants in genome-wide studies. Common practice in genetic studies is to filter variants based on population-level MAF; however, it is unclear how this applies to CH variants because it would be infeasible to compile a database that estimates the frequency of all possible combinations of *in trans* alleles in a control population. Many combinations are extremely rare, and others are private to a single individual. Furthermore, the number of possible combinations will differ by gene—many more combinations can occur in longer genes than shorter ones.

In this study, we surveyed germline CH variants in a total of 1,629 affected samples across 7 pediatric diseases: adolescent idiopathic scoliosis, congenital heart defects, disorders of sex development, ewing sarcoma, neuroblastoma, orofacial cleft, and syndromic cranial dysinnervation. Little is understood about the genetic underpinnings of these diseases (Beaty et al., 2016; Eggers et al., 2016; Grauers et al., 2016; Brohl et al., 2017; Singh et al., 2017; Tolbert et al., 2017; Pierpont et al., 2018). We used WGS data from the Gabriella Miller Kids First initiative, which has a mission of generating high-quality sequencing data for large cohorts of pediatric-disease patients and making those data available for broad use (Heath et al., 2019). For most probands, sequencing data were available for the affected child and both of her/his parents. Thus, we were able to estimate haplotypes using the best-available computational-phasing approach (Delaneau et al., 2013; Choi et al., 2018). We used computational tools to estimate pathogenicity of individual alleles within a given gene and then identified genes with multiple, potentially pathogenic alleles on homologous chromosomes. To better understand the frequency of CH variants in the general population, we estimated gene-level frequencies of CH variants in healthy controls and used these as a comparison group against our probands. Finally, we compared the observed frequencies of CH variants in each disease against the frequencies of HA variants. The resulting observations yielded novel insights about how often CH variants occur in diverse pediatric diseases, how one disease compares to another, and genes that may play a role in recessive inheritance of these pediatric diseases. This study is the first to report multi-cohort, genome-wide evaluations of CH variants in pediatric diseases.

## Materials and Methods

### Disease Cohorts

We analyzed trio data stored in the Gabriella Miller Kids First Data Resource Center (Heath et al., 2019). These data were generated via Illumina-based, WGS of non-disease cells (Gabriella Miller Kids First Pediatric Research Program, 2019) from each proband and their parents. The diseases we studied are adolescent idiopathic scoliosis (16 trios), congenital heart defects (709 trios), disorders of sex development (79 trios), Ewing’s sarcoma (287 trios), neuroblastoma (259 trios), orofacial cleft (107 trios), and syndromic cranial dysinnervation (172 trios). Specific diagnoses for each disease are in Supplementary Table 1. Trios were selected based on whether or not they were labeled as a trio in the database and DNA variant data were available for all three members of the trio. In addition, we filtered patients based on research use consent status. For all diseases except syndromic cranial dysinnervation, we used trios that had been consented for general research use or health/medical/biomedical use. For syndromic cranial dysinnervation, we used trios that were consented for disease-specific use (in accordance with data-use restrictions).

### Variant Identification for Disease Cohorts

**FIGURE 1:**
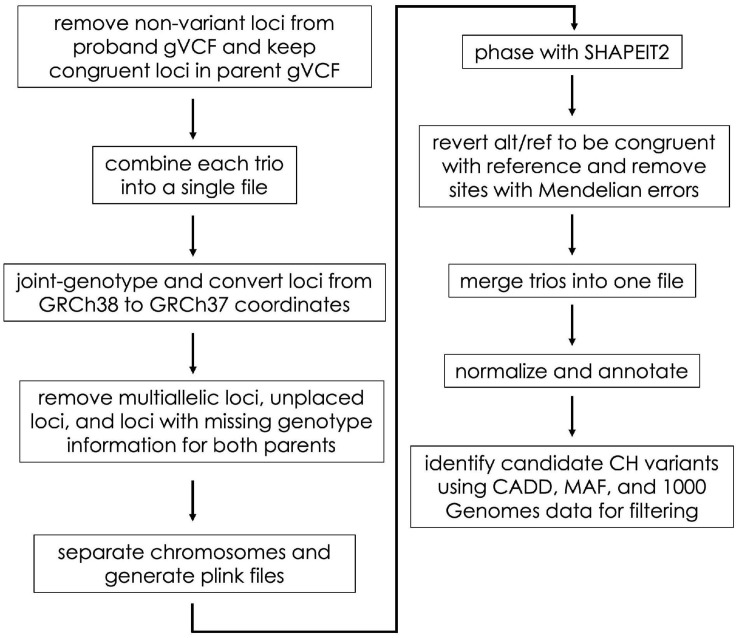
Flow diagram of the gVCF processing steps. These steps were taken prior to HA and CH variant identification.

In the Gabriella Miller Kids First Data Resource Center, germline variant data are provided as gVCF files (Poplin et al., 2017). Using these files as input, we identified CH and HA variants in a series of steps including file aggregation, liftover, phasing, annotation, and filtering (described below and in Figure 1). We completed these steps using scripts adapted from the CompoundHetVIP pipeline (Miller and Piccolo, 2020b). These and other scripts are available in an open-source repository at https://github.com/dmiller903/CompoundHetVIP-GMKF. This repository contains Python scripts^1^ with custom code for parsing metadata and handling the gVCF files in a way that is specific to the Gabriella Miller Kids First Data Resource Center. All of these scripts are stored within a Docker image, which is available at: https://hub.docker.com/r/dmill903/compound-het-vip-gmkf. A detailed example of how data from each disease was processed can be viewed at: https://github.com/dmiller903/CompoundHetVIP-GMKF/blob/master/usage_example.pdf.

For each disease, gVCF, clinical, and manifest files were downloaded from the Gabriella Miller Kids First Data Resource Center. These files were downloaded in September 2019 (adolescent idiopathic scoliosis), January 2020 (disorders sex development), February 2020 (Ewing’s sarcoma), March 2020 (congenital heart defects, orofacial cleft, syndromic cranial dysinnervation), and April 2020 (orofacial cleft). The clinical and manifest files contain information about file names, sample ID’s, family relationships, disease status, etc. The information from the clinical and manifest files were combined using the “kids_first_meta.py” script. The resulting combined file was used as input to many of the subsequent scripts to keep track of file names, family relationships, probands, etc. Unlike VCF files (Danecek et al., 2011), gVCF files contain information for variant sites as well as non-variant positions (Poplin et al., 2017). These files are large and would take much longer to process if non-variant positions were included throughout the whole pipeline. Therefore, using the “keep_variant_sites.py” script, we removed all non-variant positions for each proband (Figure 1). Using the variant positions of the proband as a reference, the script then retained the same positions in each parent’s gVCF file (regardless of whether the position was variant or non-variant).

The “combine_trios.py” script was used for each trio to combine data from all family members into a single file; we used *Genome Analysis Toolkit* (*GATK*) *(version 4.0.5.1)* for this step (Poplin et al., 2017; Figure 1). In addition, the script created a “.fam” file to be used later in processing. As part of its data-processing pipeline, the KFDRC had aligned reads to build GRCh38 of the human reference genome (Schneider et al., 2016). Alignment to this build was an issue because subsequent tools in our pipeline required the files to be aligned to GRCh37. Therefore, our “liftover.py” script joint-genotyped the combined trio files using *GATK* and converted the coordinates to GRCh37 using *Picard*’s (Picard Tools, 2019) Liftover tool. During liftover, some sites with unknown positions in the GRCh37 build were included in the VCF file; we created a “remove_unplaced_multiallelic.py” script to remove these positions. In addition, the script removed multiallelic or duplicate positions because programs such as *PLINK2* (Purcell and Chang, 2020; Chang et al., 2015) (used next) and *SHAPEIT2* (Delaneau et al., 2013) (used later) cannot handle these types of sites. For each trio, sites containing missing genotype information (i.e., “./.”) for both parents were removed to improve phasing accuracy.

*SHAPEIT2* requires chromosomes to be phased separately. PLINK files are also needed by *SHAPEIT2* during phasing. Our “separate_chr_generate_plink.py” script separated the data for each trio file into chromosome files (autosomes only) and executed *PLINK* to generate the files needed for phasing (.bed,.bim,.fam). Next, we used “phase_with_shapeit.py” to phase each chromosome. The parameters for phasing were set so that *SHAPEIT2* used family relationship genotype information and also used the 1000 Genomes Project (1000GP) phase 3 haplotype reference panel. Default parameters were used. The files used for phasing can be found at https://mathgen.stats.ox.ac.uk/impute/1000GP_Phase3.html and through the 1000GP online database (1000 Genomes Project Consortium, Auton et al., 2015).

In some cases, SHAPEIT2 switches the REF and ALT alleles during phasing. Our “alt_ref_revert.py” script ensured that the REF/ALT alleles of the phased VCF files were congruent with the REF/ALT order of the reference genome. In addition, it removed sites with Mendelian errors. To make subsequent analysis of the phased files easier, “concat_merge_phased_vcf.py” executed *bcftools* (Li, 2011) *(version 1.9)* to concatenate all phased chromosomes for each trio into a single trio file. Finally, using bcftools, each concatenated trio file was merged into a single VCF file containing all trios for a particular disease, and a.fam file was created for this merged file.

As a preprocessing step, *GEMINI* (Paila et al., 2013) recommends left trimming and normalizing VCF files using *vt tools* (Tan et al., 2015). Our “vt_split_trim_left_align.py” script used *vt tools (version 2015.11.10)* to trim and normalize the phased VCF file. Next, “annotate.py” executed *snpEff* (Cingolani et al., 2012) to annotate the variants with information about whether or not a variant is in a gene, the type of variant, the predicted effect the variant may have on protein coding (i.e., variant impact severity, and whether or not the variant causes loss of protein function), etc.

*GEMINI (version 0.30.2)* allowed us to provide additional annotations and then filter and summarize the annotated genetic variants. The “gemini_load.py” script loaded the VCF file into a GEMINI database, which provided information such as Combined Annotation Dependent Depletion (CADD) scores (Rentzsch et al., 2019) and MAF from the 1000GP (1000 Genomes Project Consortium, Auton et al., 2015) or gnomAD (Karczewski et al., 2020) (*version 2.1*). The “identify_CH_variants.py” script first exports the GEMINI database to a.tsv file and then uses that file to identify CH variants. CH variants were identified based on five criteria: (1) whether snpEff classified each of the alleles contributing to the CH variant as “HIGH” impact, (2) whether each allele had a CADD score ≥ 15, (3) neither parent was HA at either allele as a HA allele would unlikely be damaging given that it is present in a healthy parent, (4) neither parent had the exact same alleles making up a CH variant as found in the child, and (5) at least one allele of the CH variant had a MAF ≤ 5% (gnomAD MAF used as priority over 1000GP). We allowed one of the alleles to have a MAF > 5% to include scenarios where a single, damaging, common allele does not have a phenotypic effect when found to be heterozygously inherited, but may have a phenotypic effect when paired with a different, damaging, rare allele found on the opposite chromosome at a different position within the same gene. The genes in the final output are referred to as having “potentially damaging” variants.

To identify HA variants, the “identify_HA_variants.py” script was used. This script used the same GEMINI database and selected variants that were of “HIGH” impact, had a MAF ≤ 5%, and a CADD score ≥ 15. HA variants for which either parent had a HA variant at the same position as the child were excluded from the final output file.

### Identification of CH and HA Variants in 1000 Genomes Samples

Data from the 1000GP (1000 Genomes Project Consortium, Auton et al., 2015) were used as a baseline to better understand the frequency of potentially damaging CH and HA variants in the general population. Phased VCF data for the 1000GP were obtained from ftp://ftp.1000genomes.ebi.ac.uk/vol1/ftp/release/20130502/. This repository contains genotypes/haplotypes for 2,504 unrelated individuals. Because these files had previously been phased using a combination of *Beagle* (Browning and Browning, 2007) and *SHAPEIT2*, many of the steps required for the GMKF data were unnecessary. We processed chromosome-level VCF files (autosomes only) starting with the “concat_merge_phased_vcf.py” script and progressing until the “add_GDI_and_gene_lengths.py” script in the *CompoundHetVIP* pipeline (Miller and Piccolo, 2020b), a generalized version of the pipeline that was used to identify variants in the GMKF data. Because the 1000GP data does not contain trios or other family relationships, the “identify_CH_variants.py” script was not able to exclude variants based on observations in parents. However, we used the same CADD, MAF, and impact filtering criteria for the 1000 Genomes data as what we used for the GMKF variants.

### Filtering CH Variants Using 1000 Genomes Data as Reference

Using *RStudio* RStudio Team (2020) *(version 1.2.5042)*, *R (R Core Team, 2020) (version 3.6.3)* and the *tidyverse package* (Wickham et al., 2019) *(version 1.3.0)*, we further filtered the genes using potentially damaging CH variants from 1000GP as a reference. We identified genes in which a CH variant was present in more than 1% of the 1000 Genome samples and excluded these genes from the GMKF analysis. This same logic was used to filter the HA variants.

### Known Tumor Suppressor and Developmental Genes

Because inherited DNA variations in germline tissue can contribute to the inactivation of tumor suppressor genes and result in subsequent cancer development (Wang et al., 2018), we used *R* to identify which of the genes with potentially damaging CH and HA variants were in known tumor suppressor genes. In addition, given that both structural birth defects and pediatric cancers tend to occur at an early age, we identified which of the potentially damaged genes were in known developmental biology genes. During the identification process, a list of known tumor suppressor genes from Cancer Gene Census and a list of known developmental genes from Reactome (Reactome Developmental Biology pathway: R-HSA-1266738) were used (Sondka et al., 2018; Jassal et al., 2020). For these genes and all genes identified with potentially damaging CH variants, we used DisGeNET to determine whether any gene-disease associations had been identified in previous studies (Piñero et al., 2020). DisGeNET uses many different databases (e.g., UniProt, ClinVar, etc.) to establish gene-disease associations. One metric DisGeNET provides is a gene-disease association (GDA) score which ranges from 0 to 1. The more sources and publications that support a gene-disease association, the higher the value. We considered GDA values greater than or equal to 0.5 as strong evidence for a gene-disease association. In addition to GDA scores, we used DisGeNET to produce variant-disease association (VDA) scores for each potentially damaging variant contributing to a CH variant. VDA scores follow the same scale as GDA scores.

### Pathway Analysis of Genes Containing Potentially Pathogenic CH Variants

Pathway enrichment analysis can help determine whether specific biological pathways are significantly enriched based on a list of candidate genes (Reimand et al., 2019). We used the *R* and the *ReactomePA* (Yu and He, 2016) package (*version 1.30.0*), to perform a pathway enrichment analysis for each disease. Default parameters were used. For each disease, the genes with CH variants that were retained after 1000GP data filtering were used as input. Pathways that had an adjusted *p*-value (adjusted using the Benjamini-Hochberg False Discovery Rate method; Benjamini and Hochberg, 1995) less than or equal to 0.05 and a *q*-value less than or equal to 0.2 were retained.

## Results

In this study, we focus on scenarios in which at least two alternate alleles occurred on different chromosomes in the same gene and/or locus; thus, we assume the diseases follow an autosomal-recessive inheritance pattern in some cases and that these traits are subject to Mendelian inheritance. Autosomal-recessive and Mendelian inheritance have been associated with some pediatric cancer types and structural birth defects (Webber et al., 2015; Zhang et al., 2015; Miller and Piccolo, 2020a). For recessively inherited diseases, CH or HA variants may play a role if both alleles lead to loss of protein function. Therefore, we also identified HA variants, in which two non-reference alleles occurred at the same locus within a gene. We included these variants in the analysis as a way to show how the number of samples and genes with potentially damaging HA variants compares to the number of samples and genes with potentially damaging CH variants.

**FIGURE 2:**
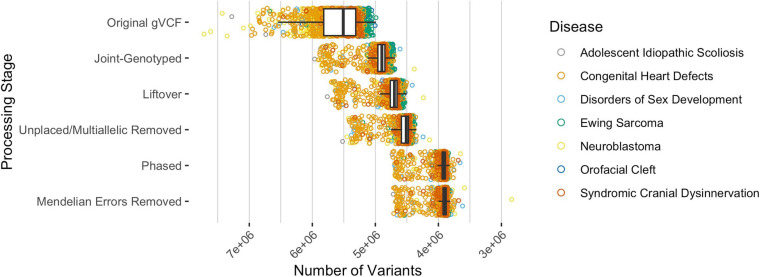
The median number of variants per sample, across all disease types, after each processing step where variants were excluded. The original gVCF files had a median of 5,509,545 unphased variants across all samples and diseases. Approximately 70.7% of the variants were available for CH and HA identification after processing the original gVCF files. Of the available variants, ∼86.7% were phased on average.

Across all analyzed pediatric diseases, the median number of unphased variants per sample was 5,509,545 prior to any data processing and variant-type classifications (Figure 2). After joint-genotyping, liftover, removing unplaced and multiallelic sites, phasing, and removing sites with Mendelian errors, the median number of unphased variants per sample was 3,894,315 (Figure 2). Therefore, on average, 70.7% of the original variants were available for CH and HA identification after these preprocessing steps. Joint-genotyping (which includes quality filtering) and phasing led to the greatest reductions in the number of unphased variants: 613,225 and 602,443 variants removed on average, respectively (Figure 2). Of the variants available for phasing, ∼86.7% were successfully phased on average. Unphased sites included those that were incompatible with the reference panel (e.g., SNP missing in reference haplotype panel), monomorphic or singleton SNPs (these sites are uninformative for phasing), and those with Mendelian inheritance errors (Delaneau et al., 2013).

**FIGURE 3:**
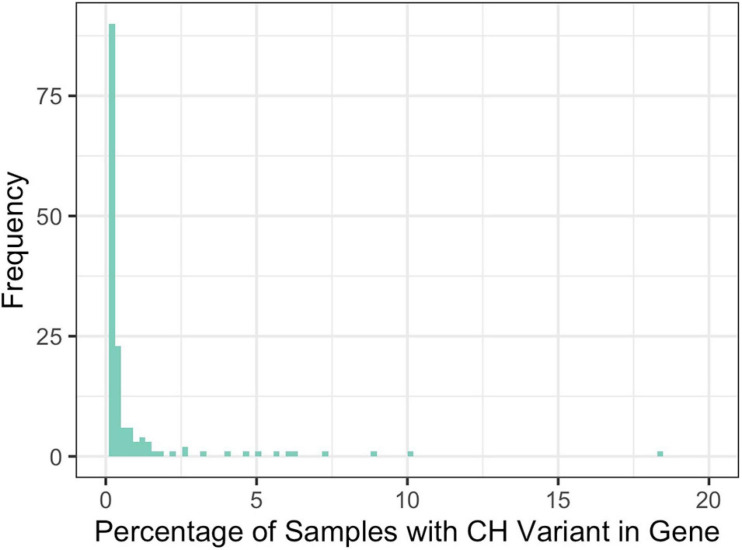
Percentage of 1000GP samples with a CH variant in a gene. Frequency represents the number of genes that were observed at a specific percentage.

Across all disease types, the initial query for CH variants resulted in 146 probands and 41 unique genes with potentially damaging CH variants that met our minimum CADD, MAF, and gene-impact thresholds during variant identification (see section “Materials and Methods”). Across all diseases, based on our initial query, an average of 9.5% of the samples had at least one CH variant and there were an average of 10.9 genes per disease with potentially damaging CH variants. To further filter these potentially damaging variants, we used 1000GP data as a baseline of what to expect, in terms of CH variation, in a seemingly healthy population. Potentially damaging CH variants were identified in 151 genes and 883 samples. Of the samples with a potentially damaging CH variant, 26 of the genes were present in more than 1% of the samples (Figure 3). For each disease population, we retained genes in which a CH variant occurred in less than 1% of the 883 1000GP samples. After this filtering step, the number of samples and genes with potentially damaging CH variants was reduced (Table 1); across all diseases, the average percentage of samples with potentially damaging CH variants decreased to 3.3% and the average number of genes per disease with potentially damaging CH variants was 5.1 (25 total unique genes, 17 of which were not identified as having HA variants). We observed similar results for HA variants before and after 1000GP filtering (Table 2). For example, across all diseases, the average number of samples with potentially damaging HA variants was 22.2% per disease and there were an average of 45 genes per disease with potentially damaging HA variants. After 1000GP filtering, the average percentage of samples with potentially damaging HA variants was 17.2% per disease, and the average number of genes with potentially damaging HA variants was 35.6 per disease.

**TABLE 1:** The number of genes and samples with potentially damaging CH variants before and after filtering with the 1000GP data.

**Disease**	**Number of genes with CH variants based on initial query**	**Number of genes with CH variants after filtering with 1000GP data**	**Number of samples with CH variants based on initial query**	**Number of samples with CH variants after filtering with 1000GP data**
Adolescent idiopathic scoliosis	3	1	2 (12.5%)	1 (6.3%)
Congenital heart defects	29	15	63 (8.9%)	18 (2.5%)
Disorders of sex development	6	2	9 (11.4%)	2 (2.5%)
Ewing sarcoma	12	6	24 (8.4%)	7 (2.4%)
Neuroblastoma	14	5	26 (10%)	5 (1.9%)
Orofacial cleft	4	3	7 (6.5%)	4 (3.7%)
Syndromic cranial dysinnervation	8	4	15 (8.7%)	6 (3.5%)

*For each disease dataset, genes that contained CH variants in less than or equal to 1% of the 1000GP samples were kept.*

**TABLE 2:** The number of genes and samples with potentially damaging HA variants before and after filtering with the 1000GP data.

**Disease**	**Number of genes with HA variants based on initial query**	**Number of genes with HA variants aftering filtering with 1000GP data**	**Number of samples with HA variants based on initial query**	**Number of samples with HA variants after filtering with 1000GP data**
Adolescent idiopathic scoliosis	7	5	4 (25%)	4 (25%)
Congenital heart defects	134	102	193 (27.2%)	129 (18.2%)
Disorders of sex development	33	24	20 (25.3%)	18 (22.8%)
Ewing sarcoma	47	39	54 (18.8%)	37 (12.9%)
Neuroblastoma	43	35	53 (20.5%)	36 (13.9%)
Orofacial cleft	19	16	19 (17.8%)	13 (12.1%)
Syndromic cranial dysinnervation	32	28	36 (20.9%)	27 (15.7%)

*For each disease dataset, genes that contained HA variants in less than or equal to 1% of the 1000GP samples were kept.*

CH variants and HA variants sometimes affect common genes, generally they occur in different genes. When researchers do not consider CH variants in genotype-phenotype studies, they may overlook important candidate genes. In turn, overlooking candidate genes may cause the investigator to overlook some samples by assuming that they have no potentially pathogenic variants when, in fact, they do. Although not our focus, this is an important factor to consider when a clinician is trying to understand what genes have contributed to a patient’s disease. Using the genes retained after filtering with 1000GP data, potentially damaging CH variants are observed in samples and genes that HA variants are not identified in. For example, all diseases showed a percent increase in the number of samples with potentially damaging CH variants that did not have potentially damaging HA variants (Table 3). In addition, all diseases experienced a percent increase in the number of genes with potentially damaging CH variants that did not have potentially damaging HA variants (Table 4).

**TABLE 3:** The number of samples with potentially damaging HA variants and the number of unique samples (not seen with potentially damaging HA variants) with potentially damaging CH variants after filtering with 1000GP data.

**Disease**	**Total samples in study**	**Total samples with HA variants after filtering with 1000GP data**	**Unique samples with CH variants after filtering with 1000GP data**	**Percent increase in the number of samples**
Adolescent idiopathic scoliosis	16	4	1	25
Congenital heart defects	709	129	11	8.5
Disorders of sex development	79	18	2	11.1
Ewing sarcoma	287	37	7	18.9
Neuroblastoma	259	36	5	13.9
Orofacial cleft	105	13	3	23.1
Syndromic cranial dysinnervation	172	27	6	22.2

**TABLE 4:** The number of genes with potentially damaging HA variants and the number of unique genes (potentially damaging HA variants not identified in gene) with potentially damaging CH variants after filtering with 1000GP data.

**Disease**	**Total number of genes with potentially damaging HA variants after 1000GP filtering**	**Number of unique genes with CH variants after 1000GP filtering**	**Percent increase in the number of potentially damaging genes**
Adolescent idiopathic scoliosis	5	1	20
Congenital heart defects	102	11	10.8
Disorders of sex development	24	2	8.3
Ewing sarcoma	39	5	12.8
Neuroblastoma	35	4	11.4
Orofacial cleft	16	3	18.8
Syndromic cranial dysinnervation	28	3	10.7

**FIGURE 4:**
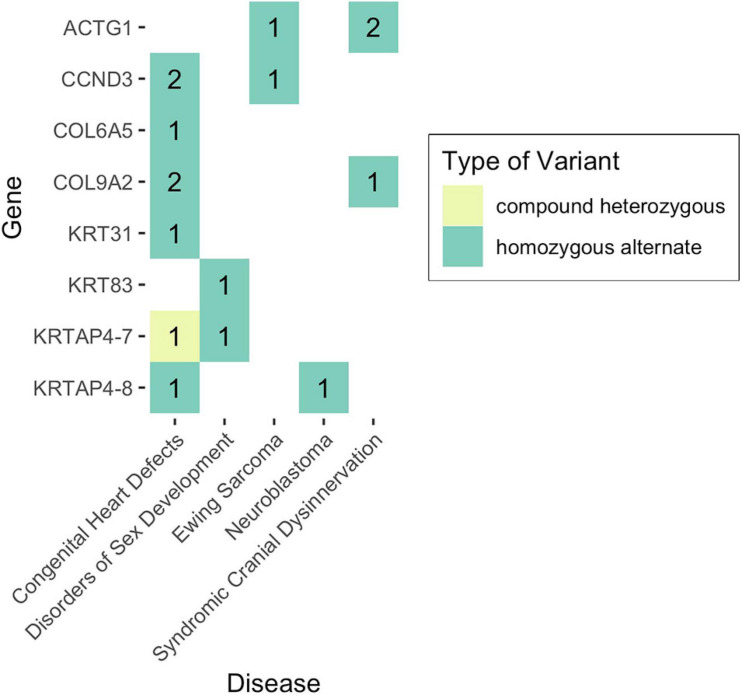
Number of samples with potentially damaging CH or HA variants in genes involved in developmental biology. No potentially damaging CH or HA variants were identified in tumor suppressor genes.

**FIGURE 5:**
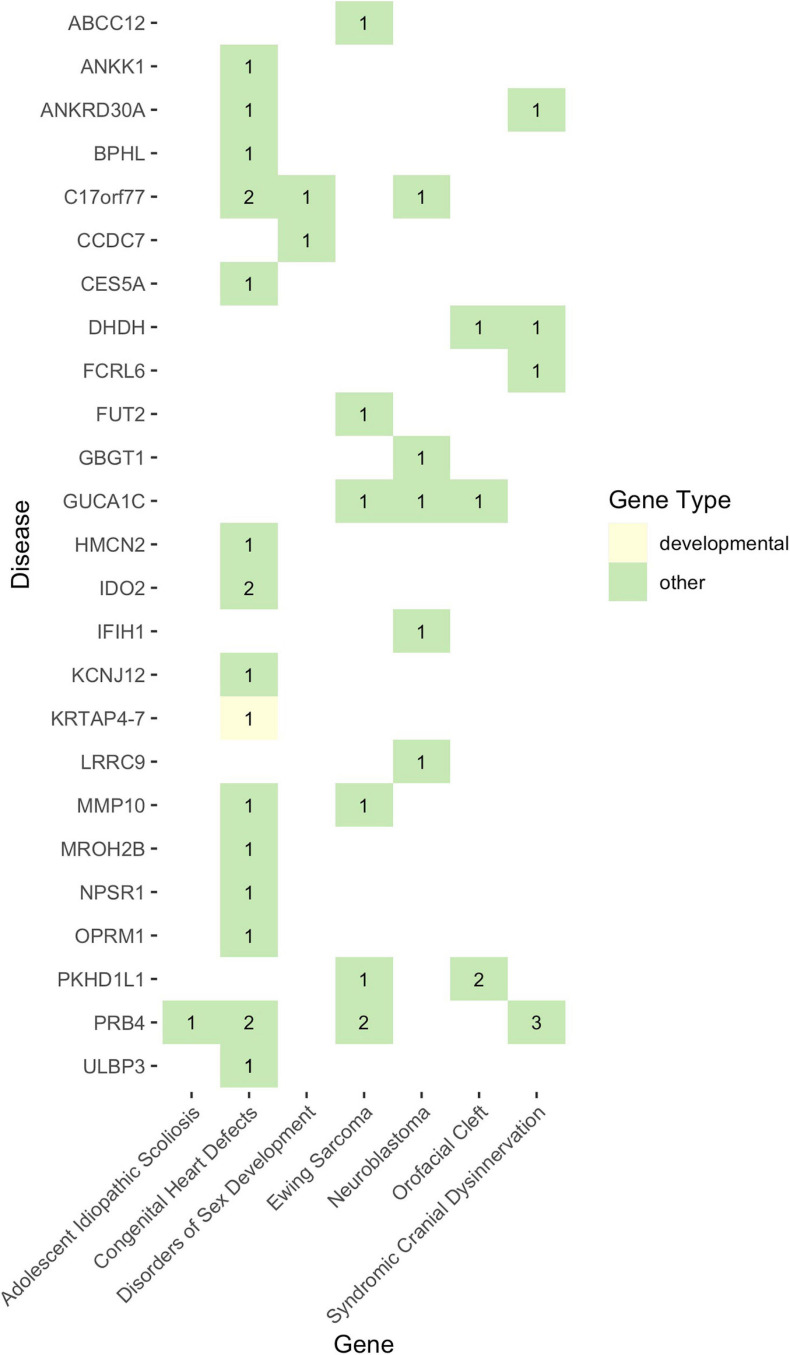
The landscape of potentially damaging CH variants. Each colored-box represents a gene type (see legend). The values within each colored-box indicate how many samples for that disease had a CH variant in that gene.

Given that non-functional tumor suppressor genes can lead to tumor development and given that pediatric cancers and structural birth defects occur early in a child’s life, we sought to determine whether any of the potentially damaged genes (after 1000GP filtering) occurred in tumor suppressor or developmental biology genes (see section “Materials and Methods”). Congenital heart defects was the only disease where a CH variant was identified in a developmental biology gene (*KRTAP4-7*: *n* = 1) (Figures 4, 5). However, *KRTAP4-7* and the variants contributing to the CH variant identified in this gene were not associated with congenital heart defects or any of the other diseases in this study based on a GDA or VDA score greater than or equal to 0.5 (Piñero et al., 2020). All diseases except adolescent idiopathic scoliosis and orofacial cleft had at least one sample with a HA variant in a gene known to be involved in developmental biology (Figure 4). No genes associated with tumor suppression were identified for either disease. Variants in the developmental biology genes *ACTG1*, *CCND3*, *COL9A2*, *KRTAP4-7*, and *KRTAP4-8* were seen in more than one disease. However, using DisGeNET, we did not identify any previous gene-disease associations between the developmental biology genes and the disease(s) they were identified in. To expand the list of candidate genes for gene-disease associations, we also analyzed all genes that contained CH variants after 1000GP filtering (Figure 5). These genes and the CH variants identified in these genes also resulted in no GDA or VDA scores greater than or equal to 0.5 for the diseases that they were identified in. Supplementary Table 2 provides a complete list of heterozygous variants that were identified as part of a CH variant, the genes and disease(s) they were identified in, and the number of samples with each heterozygous variant. Supplementary Table 3 provides a complete list of HA variants, the genes and disease(s) they were identified in, and the number of samples with each HA variant.

For each disease, we performed a pathway analysis to determine if any disease pathways were enriched with genes containing potentially damaging CH variants. Using the genes retained after 1000GP filtering of the CH variant gene data, we identified three diseases as having enriched pathways based on an adjusted p-value less than or equal to 0.05 and a q-value less than or equal to 0.2 (Table 5): Ewing sarcoma, neuroblastoma, and orofacial cleft. Each of the enriched pathways, across all diseases, had a single gene identified as being part of the pathway. All three diseases had two pathways in common: *the inactivation, recovery and regulation of the phototransduction cascade*, and *the phototransduction cascade*. These two pathways involve the *GUCA1C* gene. However, no diseases were found to have a strong association with *GUCA1C* based on a GDA search through DisGeNET. Other genes identified as being part of an enriched pathway included *FUT2*, *MMP10*, and *IFIH1*. These genes are not found in the Cancer Gene Census, nor are they part of a developmental biology pathway. Supplementary Table 4 provides all pathways identified for each disease, regardless of adjusted *p-* and *q*-values.

**TABLE 5:** Pathways enriched with genes containing potentially damaging CH variants.

**Disease**	**Enriched pathway**	**Adjusted *p*-value**	***q*-value**
Ewing sarcoma	ABO blood group biosynthesis (*FUT2*)	0.014	0.006
	Lewis blood group biosynthesis (*FUT2*)	0.028	0.013
	Blood group systems biosynthesis (*FUT2*)	0.028	0.013
	Inactivation, recovery and regulation of the phototransduction cascade (*GUCA1C*)	0.028	0.013
	The phototransduction cascade (*GUCA1C*)	0.028	0.013
	Activation of Matrix Metalloproteinases (*MMP10*)	0.028	0.013
	Collagen degradation (*MMP10*)	0.041	0.019
Neuroblastoma	Inactivation, recovery and regulation of the phototransduction cascade (*GUCA1C*)	0.018	0.008
	The phototransduction cascade (*GUCA1C*)	0.018	0.008
	Visual phototransduction (*GUCA1C*)	0.038	0.018
	NF-kB activation through FADD/RIP-1 pathway mediated by caspase-8 and -10 (*IFIH1*)	0.018	0.008
	TRAF3-dependent IRF activation pathway (*IFIH1*)	0.018	0.008
	TRAF6 mediated NF-kB activation (*IFIH1*)	0.018	0.008
	TRAF6 mediated IRF7 activation (*IFIH1*)	0.018	0.008
	Negative regulators of DDX58/IFIH1 signaling (*IFIH1*)	0.018	0.008
	Ovarian tumor domain proteases (*IFIH1*)	0.018	0.008
	DDX58/IFIH1-mediated induction of interferon-alpha/beta (*IFIH1*)	0.032	0.015
Orofacial cleft	Inactivation, recovery and regulation of the phototransduction cascade (*GUCA1C*)	0.012	0.005
	The phototransduction cascade (*GUCA1C*)	0.012	0.005
	Visual phototransduction (*GUCA1C*)	0.023	0.010

*Genes used in Pathway Enrichment Analyses are those that were retained after filtering with 1000GP data. Ewing sarcoma, neuroblastoma, and orofacial cleft were the only diseases with significantly enriched pathways (FDR adjusted p-value = 0.05 and q-value = 0.2). The gene that was identified in each pathway is in parentheses.*

## Discussion

The importance of studying CH variants in pediatric diseases has been established, but to date, the number and scope of studies that have focused on CH variants are limited. For example, most studies on CH variants in pediatric cancers have focused on a single disease type, one or a few different gene targets, and have not accounted for background rates of CH variation in diverse, healthy populations (Miller and Piccolo, 2020a). A systematic review showed that since the advent of next-generation sequencing (NGS) approximately 20 years ago, only 10 studies have used NGS to identify CH variants in pediatric cancers (Miller and Piccolo, 2020a). Of these 10 studies, seven used trios and patterns of Mendelian inheritance to infer CH variants (Valentine et al., 2014; Spinella et al., 2015; Diets et al., 2018; Diness et al., 2018; Zhang et al., 2018; Maciaszek et al., 2019; Schieffer et al., 2019), two used computational phasing without the use of trio data (Gröbner et al., 2018; Waszak et al., 2018), and one used patient RNA-seq data to confirm the presence of a single CH variant (Zhang et al., 2015). In total, these 10 prior studies examined 1,279 samples across nine different cancer types, and identified CH variants in 23 unique genes. While no comprehensive review has been published on the role of CH variation in structural birth defects to date, a search on Google Scholar for “‘structural birth defect’ AND ‘compound heterozygous’ AND ‘next generation,”’ limited to the last 20 years, uncovered four studies that used trio data and Mendelian inheritance to infer CH variants (Li et al., 2015, 2020; Takeda et al., 2017; Aarabi et al., 2018), and one study that used computational phasing without trio data (Jiang et al., 2018). These five studies included 1,192 samples across numerous structural birth defect types, and identified CH variants in eight genes likely playing a role in the formation of a defect. The computational pipelines used in these studies were inconsistent with each other, and none took background rates of CH variation in healthy populations into consideration. In this study, we analyzed two pediatric cancer types and five structural birth defect diseases for CH variants across 1,629 affected individuals using a consistent, reproducible, computational pipeline. WGS data for these individuals were phased using trio data and a haplotype reference panel. After accounting for background rates of CH variation in the 1000GP control population, we identified 25 unique genes with at least one potentially damaging CH variant.

During the variant-identification process for both CH and HA variants, we focused on variants in which both alleles were classified as having “HIGH” impact severity and thus were among the most likely to be pathogenic. Across all diseases, we identified high-impact variants that included splice-acceptor, splice-donor, start-loss, stop-gain, stop-loss, and structural-interaction variants. We focused on variants of high impact to help control for false positives. However, by excluding alleles of lower impact (which may have included missense variants, in-frame insertions or deletions, transcription-factor binding site variants, etc.), we likely excluded some true positives. Using a healthy population as a baseline may help reduce the number of false positives, as well as the number of candidate genes that must be considered in genotype-phenotype studies. Therefore, in addition to variant-level filtering, we performed gene-level filtering using 1000GP data. We observed potentially damaging CH variants in 883 out of 2,504 of these seemingly healthy individuals. For each disease that we studied, we identified genes affected with CH variants in more than 1% of 1000GP individuals and excluded these from consideration as candidate disease genes because it is unlikely that these genes are disease-causing, given their prevalence in this seemingly healthy population.

Due to the stringent variant-level and gene-level filtering that we used, this study erred on the side of specificity rather than sensitivity. However, one of our goals was to estimate a lower bound on the number of biallelic gene variants that would be missed or mischaracterized without genome phasing. Future efforts will be necessary to refine the ability to estimate pathogenicity of varying combinations of “HIGH,” “MODERATE,” and “LOW” impact alleles in the context of CH variant identification. Although we expect that the genes presented in this paper as having potentially pathogenic CH variants are among the most likely to play a role in these diseases, we likely missed other genes that should be considered. Among the genes that our filtering process excluded are *MUC19* and multiple HLA genes (*HLA-DRB5*, *HLA-B*, and *HLA-DRB1*). Mucin genes are known to be highly variant and HLA genes are among the most polymorphic genes (Akle et al., 2015; Wu et al., 2019).

Across all diseases, our analyses revealed potentially damaging CH variants in genes involved in developmental biology (Figures 4, 5), but no statistically significant gene-disease associations were identified. That is not to say that these genes have no effect on disease development. For example, *IFIH1*, for which a potentially pathogenic CH variant was identified in a single neuroblastoma patient, has been associated with lupus erythematosus (GDA score: 0.7), Aicardi-Goutieres syndrome (GDA score: 0.7), and Singleton-Merten syndrome (GDA score: 0.69) (Piñero et al., 2020). Of the genes identified in this study, further investigation into their biological role through wet lab experiments, such as gene knockdown, could reveal novel possible mechanisms of disease development.

Phasing is computationally expensive and requires additional analysis steps. However, our analysis shows that it is common to observe CH variants in diverse diseases, thus providing evidence that phasing is justified. Our CH variant identification process enabled us to identify a considerable number of potentially disease-causing variants that would not have been identified without phasing. For example, for the pediatric diseases that we studied, the number of samples with potentially damaging CH variants increased by 8.5–25.0%, and the number of genes in which these variants occurred increased by 8.3–20.0% when compared to assessing HA variants alone (Tables 3, 4). Without phased data, across all studies, 17 genes with potentially damaging variants would have been overlooked and 35 samples would have been overlooked as having potentially damaging variants.

The process that we used to identify CH variants in these cohorts has various limitations. Despite using the best available methods and tools throughout the CH variant identification process, many variant positions were removed during preprocessing steps (Figure 2). Because of this loss, additional high impact alleles may be unaccounted for, so our results may underestimate the number of genes with potentially damaging CH variants for these pediatric diseases. Much of the data loss is due to current limitations of software programs or due to data-quality issues. For example, when converting chromosomal positions from one genome build to another, the exact position must be known in the target genome (Lowy-Gallego et al., 2019). If the position is unknown in the target genome, the position will be excluded in the final output. Another software limitation that leads to data loss occurs during phasing. Phasing relies on several factors, such as the number of high-quality variant calls and completeness of a haplotype reference panel (Choi et al., 2018). Phasing completeness is also not guaranteed when using family information during phasing. For example, if both parents and the child are heterozygous at a position, or inheritance of a variant does not follow Mendelian inheritance patterns, phase may be undetermined due to these ambiguities. Across all diseases, on average, there were 14,130 variant positions per sample removed due to Mendelian inheritance inconsistencies (Figure 2). Despite these limitations, we identified many CH variants that could be explored further.

In addition to the software limitations that are part of the phasing process, phasing can be time consuming and computationally demanding. For example, phasing a single trio can take ∼4 h if only one CPU core is being used. However, using 22 CPU cores simultaneously, we reduced the duration of this process to ∼21 min per trio. Our recently developed program, *CompoundHetVIP*, can help facilitate the phasing process and assist in CH variant identification; it uses commonly used programs such as *SHAPEIT2* (for phasing) and *GEMINI* (to assist with CH variant identification) (Delaneau et al., 2013; Paila et al., 2013; Miller and Piccolo, 2020b). *CompoundHetVIP* requires minimal command-line experience. This is one example in which phasing and CH variant identification is becoming more easily accessible to researchers of all computational skill levels.

## Conclusion

Although much insight has been gained from recent studies focusing on the role of germline variants in pediatric cancer and structural birth defect diseases, many studies overlook CH variants because identification of these variants requires sequencing data from parents as well as additional time and computational resources. However, embracing these limitations is worth the additional knowledge that is gained when studying diseases that are putatively inherited in a recessive manner. Using trio data from the GMKF, across seven pediatric diseases with unknown etiologies, we showed that the number of samples and genes with potentially damaging variants increases when compared to the number of samples and genes with HA variants, alone. We used 1000GP data as a baseline of what to expect in terms of CH variation across a genome and then used this information as a filter to reduce the number of candidate genes with potentially damaging CH variants in each disease dataset. Across all seven diseases, we observed 17 genes with potentially damaging CH variants that would have been overlooked if CH variants were not considered. In addition to research applications, the identification of CH variants may be beneficial to clinicians when trying to understand what genes may be contributing to a patient’s disease.

## Data Availability Statement

Publicly available datasets were analyzed in this study. This data can be found here: Data were downloaded from https://kidsfirstdrc.org/after gaining access through dbGaP. Information on data access requests can be found for each disease as follows: Congenital Heart Data (phs001194.v2.p2); https://www.ncbi.nlm.nih.gov/projects/gap/cgi-bin/study.cgi?study_id=phs001194.v2.p2, Neuroblastoma Data (phs001436.v1.p1); https://www.ncbi.nlm.nih.gov/projects/gap/cgi-bin/study.cgi?study_id=phs001436.v1.p1, Cranial dysinnervation (phs001247.v1.p1); https://www.ncbi.nlm.nih.gov/projects/gap/cgi-bin/study.cgi?study_id=phs001247.v1.p1, Orofacial Cleft Data (phs001168.v2.p2); https://www.ncbi.nlm.nih.gov/projects/gap/cgi-bin/study.cgi?study_id=phs001168.v2.p2, Idiopathic Scoliosis Data (phs001410.v1.p1); https://www.ncbi.nlm.nih.gov/projects/gap/cgi-bin/study.cgi?study_id=phs001410.v1.p1, Disorders of Sex Development Data (phs001178.v1.p1); https://www.ncbi.nlm.nih.gov/projects/gap/cgi-bin/study.cgi?study_id=phs001178.v1.p1, and Ewing Sarcoma Data (phs001228.v1.p1); https://www.ncbi.nlm.nih.gov/projects/gap/cgi-bin/study.cgi?study_id=phs001228.v1.p1.

## Ethics Statement

Ethical review and approval was not required for the study on human participants in accordance with the local legislation and institutional requirements. Written informed consent from the participants’ legal guardian/next of kin was not required to participate in this study in accordance with the national legislation and the institutional requirements.

## Author Contributions

DM wrote the data processing scripts, processed the data, performed critical analysis, and wrote the manuscript. SP oversaw the project, and provided edits and critical feedback. Both authors contributed to the article and approved the submitted version.

## Conflict of Interest

The authors declare that the research was conducted in the absence of any commercial or financial relationships that could be construed as a potential conflict of interest.

## Footnotes

1 https://python.org
